# Clinical considerations on monkeypox antiviral medications: An overview

**DOI:** 10.1002/prp2.1164

**Published:** 2023-12-27

**Authors:** Fariba Pourkarim, Taher Entezari‐Maleki

**Affiliations:** ^1^ Student Research Committee, Faculty of Pharmacy Tabriz University of Medical Sciences Tabriz Iran; ^2^ Department of Clinical Pharmacy, Faculty of Pharmacy Tabriz University of Medical Sciences Tabriz Iran; ^3^ Cardiovascular Research Center Tabriz University of Medical Sciences Tabriz Iran

**Keywords:** brincidofovir, cidofovir, monkeypox, mpox, *Orthopoxvirus*, safety, tecovirimat

## Abstract

Monkeypox (mpox), a virus belonging to the orthopoxvirus family, can cause a zoonotic infectious disease with morbidity and cosmetic complications. Therefore, effective antiviral drugs with appropriate safety profiles are important for the treatment of patients with mpox. To date, there is no FDA‐approved drug for the treatment of mpox. However, tecovirimat, brincidofovir, and cidofovir are the candidate therapies for the management of mpox. Given the safety concerns following the use of these medications, we aimed to review evidence on the clinical considerations of mpox antiviral medications that will be useful to guide clinicians in the treatment approach. Based on the current evidence, tecovirimat has favorable clinical efficacy, safety, and side effect profile and it can be considered as first‐line treatment for mpox.

## Graphical abstract

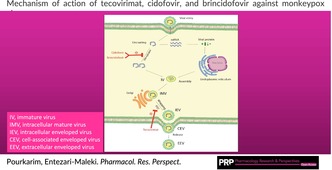

**Abbreviations**
ALT: alanine aminotransferase
AUC: area under the curve
*C*_max_
: maximum plasma concentration
CMV: cytomegalovirus
CYP: cytochrome P
EC50: concentration that inhibited virus replication by 50%
Mpox: monkeypox
MRP2: multi‐drug resistance protein 2
OATPs: organic anion transporters
UK: United Kingdom
US: United States

## 1 INTRODUCTION

Mpox virus is a virus from the orthopoxvirus genus belonging to the Poxviridae family which can cause a zoonotic infectious disease with both animal‐to‐human and human‐to‐human transmissions.^1^ In the 1970s, the first case of mpox in humans was detected in the Democratic Republic of the Congo, with other outbreaks in Africa, the United States (US), and Europe.^2^, ^3^, ^4^, ^5^ In May 2022, a cluster of mpox cases was reported in non‐endemic countries, including the United Kingdom (UK), Canada, and Australia.^6^, ^7^, ^8^ During the current outbreak (1 January 2022 to 30 September 2023), 91 123 laboratory‐confirmed cases of mpox and 157 deaths were reported to WHO from 115 affected countries.^9^ Clinical manifestations of mpox are fever, swollen lymph nodes, myalgia, back pain, headache, and skin rash.^10^ The real‐time reverse‐transcriptase‐polymerase chain reaction method is the most commonly used test for the confirmation of mpox.^11^ Due to the morbidity and cosmetic concerns such as permanent skin scars and lasting corneal scaring^12^ regarding mpox infection, effective pharmacotherapy is very important to prevent the disease complications. Although there is still no FDA‐approved drug for the treatment of mpox infection, the anti‐smallpox medications, including tecovirimat, cidofovir, and brincidofovir, are the candidate therapies for the management of mpox. However, there are several concerns about the use of these medications. For example, the results of a clinical pharmacokinetics study conducted by Cundy^13^ showed that the administration of cidofovir can be associated with dose‐limiting nephrotoxicity. Chittick et al.^14^ reported that treatment with brincidofovir may be related to increased liver enzymes and hepatic dysfunction. Also, neutropenia and increased risk of infections in mpox patients have been reported with cidofovir in clinical trials.^15^

Given the safety concerns following the use of mpox medications, we aimed to review the current evidence regarding clinical considerations, drug–drug interactions, and adverse drug events of these medications in the treatment of patients with mpox. To the best of our knowledge, this review is the first one that looks at the evidence regarding the clinical concerns of mpox medications.

## 2 SEARCH METHOD

Electronic searches were performed in PubMed, Scopus, and Google Scholar using the key terms of monkeypox, mpox, monkeypox treatments, tecovirimat, TPOXX, ST‐246, CMX001, TEMBEXA, cidofovir, brincidofovir, tecovirimat drug–drug interactions, cidofovir drug–drug interactions, and brincidofovir drug–drug interactions, and a total of 63 publications were included in this review. Non‐English written articles were excluded from the search.

## 3 TECOVIRIMAT

**FIGURE 1:**
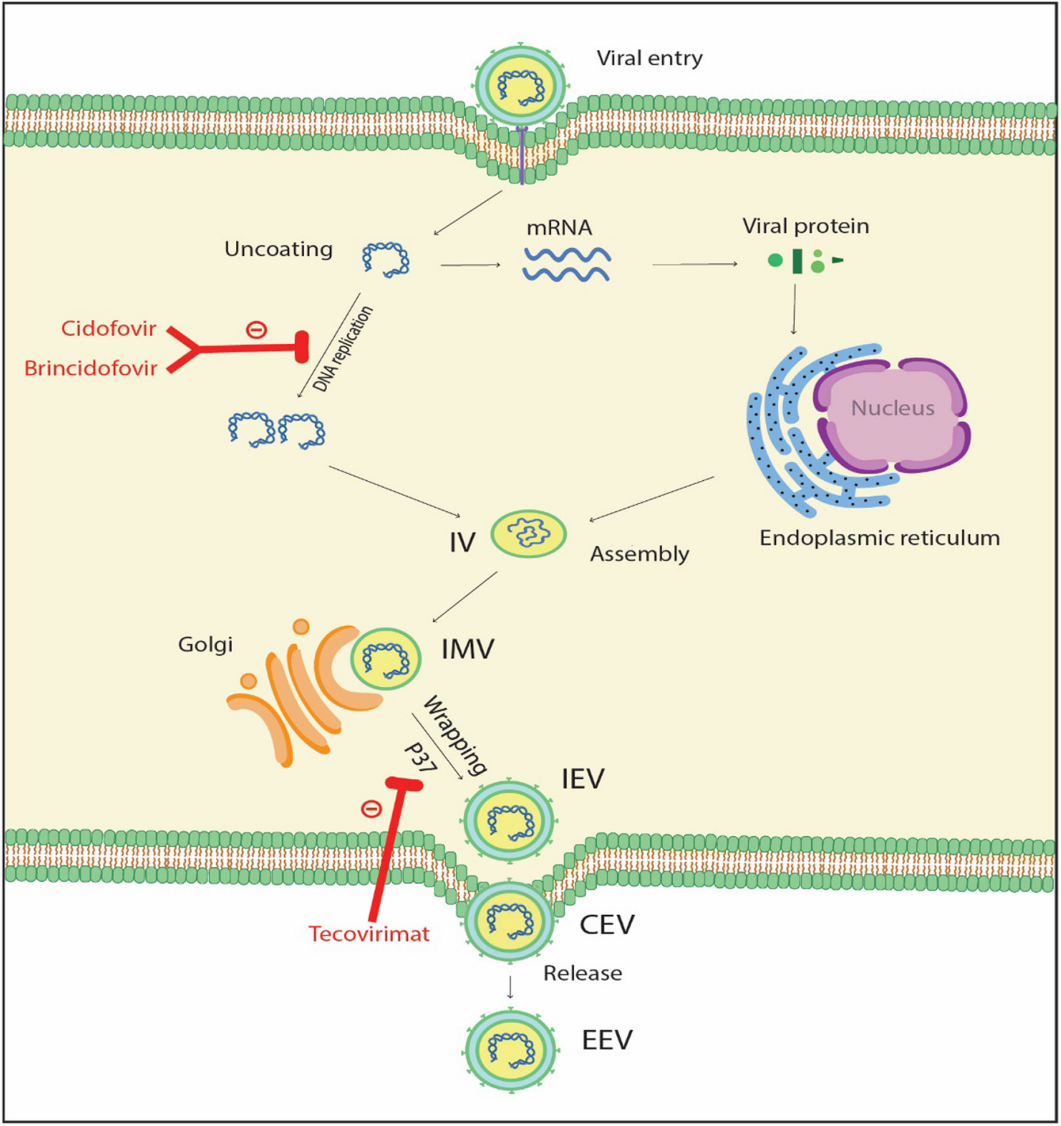
Molecular mechanism of action of tecovirimat, cidofovir, and brincidofovir in mpox. CEV, cell‐associated enveloped virus; EEV, extracellular enveloped virus; IEV, intracellular enveloped virus; IMV, intracellular mature virus; IV, immature virus.

Tecovirimat (TPOXX, ST‐246), a low‐molecular‐weight antiviral drug, is the first FDA‐approved treatment for smallpox treatment in adults and pediatrics weighing ≥13 kg.^16^, ^17^ Tecovirimat has been licensed for the treatment of mpox in the European Union and the US.^18^ It inhibits orthopoxvirus replication by inhibiting the p37 envelope protein.^19^, ^20^ The details of the molecular mechanism of action of tecovirimat are shown in Figure 1. Animal studies showed that tecovirimat could inhibit a range of other viruses including vaccinia, ectromelia, cowpox, variola, and rabbitpox.^21^, ^22^, ^23^, ^24^ Sbrana et al.^25^ investigated the prophylactic effect of tecovirimat in a subcutaneous mpox model. In this study, tecovirimat 100 mg/kg was given within 0–4 days after infection for 14 days and demonstrated 100% protection against mpox. Several studies evaluated the prophylactic effects of tecovirimat in the intravenous mpox model. Tecovirimat with doses of 3–300 mg/kg demonstrated 100% protection if administered up to 5 days postinfection. In a study with a duration of 14 days; 3 mg/kg was the effective dose, but the dose of 10 mg/kg also decreased viremia and lesion count.^26^, ^27^, ^28^ Administration of tecovirimat with food may significantly increase its absorption with the maximum plasma concentration (*C*_max_) and area under the curve (AUC)_0–24_ increasing about 45% at a steady state.^27^, ^29^ The most common adverse effects experienced with tecovirimat are headache and nausea.^30^ Based on the pivotal study, the rate of drug adverse events leading to discontinuation of tecovirimat was very low (1.7%).^30^ Also, the clinical safety study showed that tecovirimat at a dose of 600 mg orally twice daily for 14 days is safe. Thus, tecovirimat may be a good option for the treatment of patients with mpox infection due to the favorable clinical efficacy, safety, and side effect profile. Tecovirimat is a weak cytochrome P450 (CYP) 3A4 inducer and a weak inhibitor of CYP2C19 and CYP2C8.^30^ It may increase the serum levels of repaglinide in diabetic patients via CYP2C8 inhibition which can result in hypoglycemia, thus monitoring of blood glucose and hypoglycemia symptoms is necessary. It also may decrease the serum concentration of midazolam and may require dose adjustment or alternative sedatives. Coadministration of tecovirimat with QT‐prolongation agents (such as class I or class II antiarrhythmics) may increase the risk of long QT syndrome and should be avoided.^32^ Tecovirimat may decrease the serum level of tacrolimus and sirolimus via induction of CYP3A4. Thus, close monitoring of therapeutic levels of these immunosuppressive drugs is recommended in solid organ transplant recipients receiving tacrolimus or sirolimus with tecovirimat. It also may reduce the serum level of hormonal contraceptives by induction of CYP3A4 but there was no report on drug‐related pregnancies in clinical trials.^30^ However, the patient should use an alternative method of contraception during the co‐administration of tecovirimat with hormonal contraceptives and for 28 days after tecovirimat discontinuation. Due to the decreasing efficacy of tecovirimat in obese and immunocompromised patients, it should be used with close monitoring in these populations. Animal studies have not shown that tecovirimat has embryotoxic and teratogenic effects.^31^ Tecovirimat has not been evaluated in breastfeeding and data are not available in this population therefore it was not recommended for use during the breastfeeding period.^32^ Clinical considerations of tecovirimat are summarized in Table 1.

**TABLE 1:** Clinical considerations of mpox antiviral medications.

Medication	Administration	Drug–drug interactions	Pregnancy	Lactation	Adverse effects and management
Tecovirimat	Food may significantly increase its absorption (45% increase in steady state)	Repaglinide: ↑ serum levels of repaglinide Management: monitor blood glucose and hypoglycemia symptoms	It is safe and its use is recommended	Lack of data Not recommended	Nausea and headache Management: Administer with food to minimize the adverse effects
		Midazolam: ↓ serum level of midazolam Management: dose adjustment or alternative sedatives			
		Tacrolimus and sirolimus: ↓ serum level of tacrolimus and sirolimus Management: close monitoring of the therapeutic blood levels of tacrolimus and sirolimus			
		Hormonal contraceptives: ↓ serum level of hormonal contraceptives Management: use an alternative method of contraception during and for 28 days after tecovirimat discontinuation			
Brincidofovir	Oral brincidofovir should be administered on an empty stomach as food decreases its *C*_max_ and AUC	Cabozantinib: ↑ plasma concentration of cabozantinib Management: Monitor therapy	Contraindicated	Lack of data Not recommended	Diarrhea (the most frequent dose‐limiting adverse effect), nausea, vomiting, and abdominal pain Management: administer with a low‐fat meal and limit doses to 200 mg/week or less Elevated ALT Management: consider discontinuation if ALT levels are persistently greater than 10 times the upper limit of normal
		Cladribine: ↓ therapeutic effect of cladribine Management: avoid combination			
		Corticosteroids, methotrexate, and immunosuppressants (such as cyclosporine): ↓ therapeutic effect of brincidofovir Management: monitor therapy			
		OATP1B1/1B3 inhibitors, such as rifampin, clarithromycin, erythromycin, and gemfibrozil: ↑ level of brincidofovir and ↑ brincidofovir adverse effects Management: consider alternatives to OATP1B/1B3 inhibitors for the treatment of patients treated with brincidofovir or these drugs should be taken at least 3 h apart.			
		Live smallpox and mpox vaccines: ↓ protective effect of vaccines Management: monitor therapy			
Cidofovir	To minimize the risk of nephrotoxicity, premedication with probenecid prior to and 8 h after infusion of cidofovir is recommended. Double gloving, a protective gown, and ventilated engineering controls are required during the administration of cidofovir	Cabozantinib: ↑ plasma concentration of cabozantinib Management: monitor therapy	Contraindicated	Lack of data Not recommended	Nephrotoxicity (the main dose‐limiting toxicity) Management: consider appropriate hydration and the use of probenecid 2 g 3 h prior to the cidofovir, then 1 g 2 h, and 8 h after the cidofovir infusion completion
		Cladribine: ↓ therapeutic effect of cladribine Management: avoid combination			Neutropenia Management: check neutrophil count at baseline and during cidofovir therapy
		Tenofovir: ↑ plasma levels of cidofovir and enhance its nephrotoxicity Management: monitor therapy			Hypotony and uveitis management: obtain monthly ophthalmologic exam

## 4 BRINCIDOFOVIR

Brincidofovir (CMX001, TEMBEXA), an analog of cidofovir, is converted to cidofovir and inhibits orthopoxvirus DNA polymerase‐mediated viral DNA synthesis (Figure 1).^33^ It was granted fast‐track designation and Orphan Drug Status for the treatment of smallpox in June 2018.^34^ It has activity against other DNA viruses such as adenovirus, BK virus, and cytomegalovirus (CMV). Based on limited studies in animal models, oral administration of brincidofovir is effective in the treatment of mpox animal infection model^35^ but the use of brincidofovir (200 mg once weekly orally) in three human patients with mpox has resulted in no clinical benefit to patients.^14^ Diarrhea is the most frequent dose‐limiting adverse effect associated with brincidofovir (reported in approximately 70% of patients) and may be often serious (in 33% of patients).^36^, ^37^ Thus, monitoring of diarrhea and dehydration symptoms in patients under treatment with brincidofovir is recommended. Also, the dose of brincidofovir should be limited to 200 mg/week or less for the prevention of diarrhea.^36^, ^37^ Nausea, vomiting, and abdominal pain are additional gastrointestinal‐reported adverse effects of brincidofovir.^36^ Oral brincidofovir should be administered on an empty stomach as food decreases its *C*_max_ and AUC but it can be taken with a low‐fat meal to decrease gastrointestinal adverse effects. Elevated alanine aminotransferase (ALT) is a common finding seen during treatment with brincidofovir. Thus, liver function tests should be obtained before initiating and during treatment with brincidofovir, and discontinuation of brincidofovir should be considered if ALT levels are persistently greater than 10 times the upper limit of normal.^38^ Brincidofovir has less kidney toxicity than cidofovir because it is not a substrate of the human organic anion transporters (OATPs) and hence, it does not accumulate in renal tubules.^36^ It is recommended that brincidofovir should not be administered with intravenous cidofovir because brincidofovir is converted to cidofovir and increases the risk of nephrotoxicity.^38^ The reproductive study showed that brincidofovir is embryotoxic in rabbits and therefore contraindicated in pregnancy.^36^ It has not been assessed in breastfeeding and data are not available in this population. However, breastfeeding is not recommended in patients with mpox, due to the potential risk of virus transmission through direct contact between the mother and breastfed infant.^38^ It is also considered a probable human carcinogen and mutagen and may decrease male fertility via the reduction in sperm motility and effect on mitotic spermatogenesis.^38^ Brincidofovir is a substrate of OATP1B1/1B3 transporters and inhibits the multi‐drug resistance protein 2 (MRP2). It may increase the plasma concentration of cabozantinib by MRP2 inhibition.^39^ Brincidofovir may decrease the therapeutic effect of cladribine via intracellular phosphorylation and the combination of these drugs should be avoided. Immunosuppressants such as cyclosporine may reduce the effect of brincidofovir.^40^ Corticosteroids and methotrexate may reduce the therapeutic effect of brincidofovir. OATP1B1/1B3 inhibitors, such as rifampin, cyclosporine, clarithromycin, erythromycin, and gemfibrozil may elevate brincidofovir levels and increase brincidofovir adverse effects. Therefore, the clinician should consider alternatives to OATP1B/1B3 inhibitors for the treatment of patients treated with brincidofovir or these drugs should be taken at least 3 h apart.^40^ Brincidofovir may decrease the protective effect of live smallpox and mpox vaccines via the reduction of the immune response to the vaccine. Clinical considerations of brincidofovir are summarized in Table 1.

## 5 CIDOFOVIR

Cidofovir, a cytidine nucleotide analog of cytosine, is converted to an active metabolite (cidofovir diphosphate) by the host‐cell enzymes and inhibits the viral DNA polymerase (Figure 1). It has potent activity against CMV, herpes simplex virus, varicella‐zoster virus, and Epstein–Barr virus.^41^, ^42^, ^43^ The effectiveness of cidofovir in the treatment of lethal mpox infection in animal models has been demonstrated, but the evidence for cidofovir efficacy against human mpox infection is lacking.^13^, ^44^ Cidofovir was dosed for mpox as 5 mg/kg intravenously once weekly for 2 weeks, followed by 5 mg/kg IV once every other week.^45^ Animal studies showed that cidofovir is carcinogenic. Based on the National Institute for Occupational Safety and Health (NIOSH) recommendation, double gloving, a protective gown, and ventilated engineering controls are required during the administration of cidofovir. The most common adverse effects of cidofovir include proteinuria, nephrotoxicity, neutropenia, hypotony of the eye, uveitis, and infection.^13^ Nephrotoxicity is the main dose‐limiting toxicity of cidofovir. Therefore, consideration must be given toward the appropriate hydration and the use of probenecid 2 g 3 hours prior to the cidofovir dose, then 1 g 2 h and 8 h after completion of the cidofovir infusion.^46^ To reduce the risk of nephrotoxicity associated with cidofovir, serum creatinine and urine protein should be closely monitored 48 hours prior to each dose of cidofovir, and dose adjustment or discontinuation of therapy may be required depending on the degree of renal impairment.^15^ Also, nephrotoxic medications, such as amphotericin B, pentamidine, cyclosporine, contrast media, tacrolimus, nonsteroidal anti‐inflammatory drugs, and aminoglycosides, should be avoided during and at least 7 days before the initiation of cidofovir.^47^ The neutrophil count should be checked at baseline and during cidofovir therapy because neutropenia has been reported in approximately 20% of patients in clinical trials.^15^ A monthly ophthalmologic exam of the retina is necessary because hypotony and uveitis have also been reported following cidofovir therapy.^15^ Other adverse effects include nausea, vomiting, dyspnea, and Fanconi syndrome. Contraindications for the use of cidofovir include serum creatinine >1.5 mg/dL, calculated creatinine clearance ≤55 mL/min, proteinuria ≥2+, co‐administration with nephrotoxic medications, and hypersensitivity to cidofovir.^15^ Cidofovir is embryotoxic and teratogenic, and should not be used in pregnant women. It may consider only in critically ill pregnant patients who failed to respond to tecovirimat.^31^, ^48^ Due to the potential risk of cidofovir for serious adverse effects in breastfeeding infants, breastfeeding is not recommended.^46^ Cidofovir inhibits MRP2 and may increase the plasma concentration of cabozantinib.^39^ Cidofovir may diminish the effect of cladribine via intracellular phosphorylation; thus, the coadministration of these drugs should be avoided.^45^ Zidovudine plasma levels may increase when coadministered with probenecid, therefore, it is recommended that zidovudine should be discontinued or administered with 50% dose on the day of cidofovir infusion.^15^ Tenofovir may increase the plasma levels of cidofovir and enhance its nephrotoxicity. Clinical considerations of cidofovir are summarized in Table 1.

## 6 DRUG RESISTANCE

Drug resistance is a clinical concern with mpox drugs but its risk is relatively low. In vitro studies have identified a cowpox virus variant resistant to tecovirimat as a result of a mutation in the *V061* gene of the virus (homologous to variola *F13L* gene) that encodes for the p37 protein. Therefore, in vitro concentration that inhibited virus replication by 50% (EC50) for these resistant variants (>40 μM) was higher (more than 800‐fold) compared with the wild‐type cowpox virus (0.050 μM).^19^

Lederman et al. reported tecovirimat resistance in a patient with acute myelogenous leukemia under treatment with oral (200 mg) and topical tecovirimat following ACAM2000‐induced progressive vaccinia.^49^ Brincidofovir was added to the treatment at a dose of 700 mg weekly. The treatment course included 73 days of oral tecovirimat (75 g), 68 days of topical tecovirimat, and 6 weeks of brincidofovir.^49^ Unlike brincidofovir, resistance to tecovirimat was detected following prolonged subtherapeutic levels and concurrent use of topical formulation. Variation in several single nucleotide sites such as T289A, K174N, S215F, P243S, T245I, Y285H, R291K, and D301del was detected in the sequenced *F13L* gene region after tecovirimat use without a clear resistance pattern.^49^, ^50^, ^51^ Immunocompromised conditions such as AIDs, and prolonged monotherapy with tecovirimat are the main factors for tecovirimat resistance.^50^, ^51^

## 7 PRECLINICAL DRUG CANDIDATES WITH ANTIVIRAL EFFICACY AGAINST ORTHOPOXVIRUSES

Based on the in vivo studies, the effectiveness of several compounds against Orthopoxviruses has been demonstrated. However, further clinical trial studies are required to confirm their therapeutic effects in patients with mpox. NIOCH‐14, a prodrug of tecovirimat, is a P37 inhibitor that showed antiviral effects against Orthopoxviruses in in vitro and in vivo studies. Oral NIOCH‐14 has been shown good efficacy in mice and marmots infected with mpox when administered 1 day before or 2 h after infection at a dose of 10 mg/g for 6 days.^52^ A hypothesis proposed that Orthopoxviruses required tyrosine kinases for the formation of actin tail and tyrosine kinase inhibitors may block the release of viruses from the cell. In vivo study in the mice infection model showed that the preinfection uses of imatinib mesylate at a dose of 200 mg/kg once daily led to a survival rate of 100%. Also, a significant improvement in the survival rate of mice models was observed when imatinib was administered on the infection day or 1 day after infection.^53^ Mitoxantrone revealed promising antipoxvirus activity in cell culture but the in vivo study showed no beneficial effect in mice model.^54^ Administration of subcutaneous ribavirin at 100 mg/kg once daily for 5 days in mice models infected with 3 × 10^5^ plaque‐forming unit (PFU) of cowpox virus, resulted in a survival rate of 100% compared with the placebo group, in which all mice died. However, it has no significant survival benefit in mice infected with a high dose (≥3 × 10^6^ PFU) of the virus.^55^

Also, several investigational agents including adamantane derivatives, monoterpenoid derivatives, PAV‐866 and its derivatives, resveratrol, interferon‐β, and tiazofurin have shown promising antiviral effects in in vitro studies.^56^ Adamantane derivatives showed an antiviral effect against the vaccinia virus via P37 inhibition in molecular docking and in‐vitro studies.^57^ Monoterpenoid derivatives such as camphor and borneol derivatives revealed antiviral activity against variola and vaccinia. However, evidence regarding the efficacy of these derivatives on mpox is lacking.^58^ PAV‐866 is a methylene blue analog with in‐vitro antiviral activity against mpox and cowpox viruses through the inactivation of virions before infection and inhibition of binding, fusion, and entry of the virus.^59^ Cao et al.^60^ studied resveratrol efficacy on mpox in HeLa cells. The results of this study showed that 50 μmolar resveratrol can decrease the yield of mpox‐WA and mpox‐ROC clades by 195‐ and 38‐fold, respectively. Resveratrol has been shown to decrease mpox replication in comparable amounts to the well‐known Orthopoxvirus inhibitor, AraC. The prophylactic and therapeutic effect of interferon‐β against mpox in Hela cells was investigated by Johnston et al.^61^ The results showed that treatment with interferon‐β can significantly decrease mpox production and spread. Also, pretreatment with high‐dose interferon‐β (>1000 U/mL) 24 h before infection showed a 99% decrease in viral titers. The suggested mechanism for the antiviral effects of interferon‐β is the induction of MxA protein expression. Tiazofurine showed an inhibitory effect on mpox and variola virus via inhibition of inosine monophosphate dehydrogenase.^55^ However, data regarding their efficacy in in‐vivo studies is lacking.

## 8 FUTURE PERSPECTIVE

The recent outbreak of mpox revealed that cutaneous contact is not only transmission route of the virus but also can be transmitted via biologic fluids.^62^ Also, mpox mutations may increase the virus virulence and lead to a higher prevalence in future outbreaks. On the other hand, current mpox drugs are related to concerns including adverse drug effects (such as nephrotoxicity and hepatotoxicity) and risk of resistance (mostly with tecovirimat). Therefore, further clinical trials are required to investigate repurposed therapeutics and introduce safe and effective antiviral drugs with less resistance risk to the market.

## 9 CONCLUSION

In May 2022, mpox emerged in several countries, including the UK, Canada, and Australia, and has rapidly speared to other countries with increasing reported cases. There is no FDA‐approved drug for the treatment of mpox. However, antiviral agents used for the treatment of smallpox, such as tecovirimat, brincidofovir, and cidofovir can be effective in mpox. Based on the current evidence, tecovirimat has favorable clinical efficacy, safety, and side effect profile and it can be considered first‐line treatment for mpox. We summarized published literature on these agents' safety, efficacy, clinical considerations, adverse effects and drug–drug interactions and it will be useful to guide clinicians in the treatment approach.

## AUTHOR CONTRIBUTIONS

Fariba pourkarim: Investigation and writing—original draft. Taher Entezari‐Maleki: Conceptulization, writing—review and editing and supervision.

## FUNDING INFORMATION

None.

## DISCLOSURE

The authors declare that there is no conflict of interest.

## ETHICS APPROVAL STATEMENT

No ethical approval is required for the review article.

## Data Availability

Not applicable.
